# Local production of CCL3, CCL11, and IFN-γ correlates with disease severity in murine parainfluenza virus infection

**DOI:** 10.1186/1743-422X-10-357

**Published:** 2013-12-21

**Authors:** Manika Suryadevara, Cynthia A Bonville, Helene F Rosenberg, Joseph B Domachowske

**Affiliations:** 1 Department of Pediatrics, SUNY Upstate Medical University, 750 East Adams Street, Syracuse, NY 13210, USA; 2 Laboratory of Allergic Diseases, National Institute of Allergy and Infectious Diseases, National Institutes of Health, Bethesda, Maryland 20892, USA

**Keywords:** Sendai virus, Parainfluenza virus, CCL3, MIP-1α, CCL11, Eotaxin, Interferon-γ

## Abstract

**Background:**

Using a murine model of parainfluenza virus infection (mPIV1 or Sendai virus; SeV), we compared the inflammatory responses to lethal and sub-lethal infections in inbred DBA/2 mice.

**Methods:**

Mice were intranasally inoculated with either 1.6×10^3^ or 1.6×10^5^ infectious units (IU) of SeV or diluent control. Clinical data including daily weights, oxygen saturation, and lung function via whole body plethysmography were collected on days 0, 3–7, and 9–14. Clarified whole lung homogenates were evaluated for inflammatory markers by enzyme-linked immunoassay (ELISA). Data were analyzed using ANOVA or Student t-tests, as appropriate.

**Results:**

Mice inoculated with 1.6×10^5^ IU of SeV developed a lethal infection with 100% mortality by day 7, while mice inoculated with 1.6×10^3^ IU developed a clinically significant infection, with universal weight loss but only 32% mortality. Interestingly, peak virus recovery from the lungs of mice inoculated with 1.6×10^5^ IU of SeV did not differ substantially from that detected in mice that received the 100-fold lower inoculum. In contrast, concentrations of CCL5 (RANTES), CCL11 (eotaxin), interferon-γ, CXCL10 (IP-10), and CCL3 (MIP-1α) were significantly higher in lung tissue homogenates from mice inoculated with 1.6×10^5^ IU (p < 0.05). In the lethal infection, levels of CCL11, interferon- γ and CCL3 all correlated strongly with disease severity.

**Conclusion:**

We observed that severity of SeV-infection in DBA/2 mice was not associated with virus recovery but rather with the levels of proinflammatory cytokines, specifically CCL11, interferon- γ and CCL3, detected in lung tissue in response to SeV infection.

## Background

Human parainfluenza viruses (hPIV), of the *Paramyxovirus* family, cause a spectrum of illness from mild upper respiratory infection and otitis media to severe laryngotracheobronchitis and bronchiolitis [1-4]. HPIV can be detected in up to 30% of children hospitalized for acute respiratory tract infection, second in etiology of infection only to respiratory syncytial virus (RSV) [1-4]. Pathogenesis of hPIV is believed to include both direct virus cytotoxicity and subsequent host immune response; treatment with glucocorticoids provides clinical benefit in some circumstances [5-7].

Airway epithelium infected with hPIV produces a variety of cytokines and chemokines that act as immune mediators in response to infection [8]. We have previously reported increased concentrations of interleukin-6 (IL-6), CCL5 (regulated and normal T cell expressed and secreted (RANTES)), CXCL8 (interleukin-8), CXCL9 (monokine induced by gamma-interferon (MIG)), and CCL3 (macrophage inflammatory protein-1α (MIP-1α)) from the nasal wash specimens of children infected with hPIV when compared to uninfected controls [9]. These cytokines contribute to the recruitment of inflammatory cells to the infected epithelium and, in the case of CXCL8, is associated with illness severity [9].

Sendai virus (SeV), murine PIV, induces acute bronchiolitis and interstitial pneumonia in rodents, and has been used to model severe human hPIV infection [10-12]. Differences in susceptibility to SeV infection among mouse strains exist, with C57BL/6 mice being more resistant and DBA/2 mice being more susceptible to infection [11,13,14]. SeV is known to be a strong inducer of various cytokines/chemokines, including interferon-Υ, IL-2, TNF-α, IL-6, and IL-10 [12]. Simon and colleagues showed that SeV infection in the susceptible DBA/2 mice resulted in a vigorous inflammatory response, specifically with increased production of IL-1β, IL-2, IL-6, interferon-γ, and TNF-α, with subsequent mortality from severe lung injury. Similarly, in comparison to the resistant C57BL/6 mice, up-regulation of CCL3 and CCL11 was seen in the SeV-infected DBA/2 mice [11].

Understanding host inflammatory responses that contribute to illness severity offers the potential to identify future therapeutic targets. Toward this end, we compared the inflammatory responses to both sub-lethal and lethal SeV infection in mice.

## Results

### Clinical parameters of SeV-infected mice

**Figure 1:**
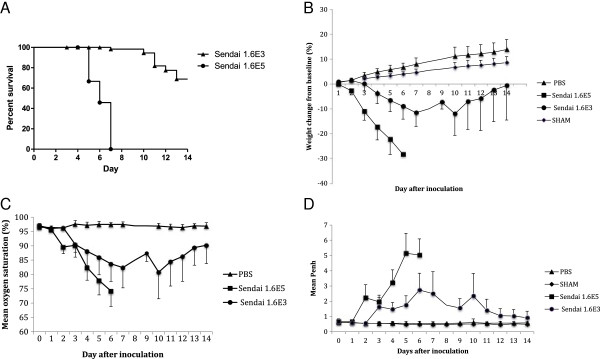
**Clinical parameters. (A)** Survival curve, **(B)** Mean weight change from baseline (%), **(C)** Mean oxygen saturation (%), **(D)** Mean enhanced pause (Penh) as measured by whole body plethysmography. PBS: phosphate buffered saline (control). SHAM: sham stock, prepared from uninfected mouse lung homogenate. In **(A)**, there is statistical difference between the two survival curves (p < 0.05). In **(B)**, **(C)**, and **(D)**, at all time points day 3 and later, clinical parameters of SeV-infected mice differed significantly from control mice inoculated with either PBS or sham stock (p < 0.05 via ANOVA analysis). There is no data for the mice inoculated with 1.6x10^5^ IU of SeV after day 5 due to death of the mice. 1.6E5 and 1.6E3: 1.6×10^5^ and 1.6×10^3^ IU of SeV inoculated in 50 μl, respectively. Error bars represent standard deviation.

Mice inoculated with 1.6x10^3^ IU of SeV developed a clinically significant infection with 32% mortality in the 14-day period (Figure 1A, p < 0.05). When compared to control mice, the infected mice showed changes in weight (p < 0.05), oxygen saturations (p < 0.05), and Penh values (p < 0.05). Clinical symptoms in the infected mice peaked between days 6 and 10, with nadir mean weight change from baseline of -12%, nadir mean oxygen saturation of 81%, and peak Penh values at 4-fold over baseline. Following these points, clinical parameters were noted to improve, although symptoms did not resolve completely; by day 14, mean oxygen saturations of infected mice remained statistically lower than those of control mice, and mean Penh values in infected mice remained at 1.5-fold over baseline (Figure 1).

Mice inoculated with 1.6×10^5^ IU of SeV developed a lethal infection, with 100% mortality by day 7. These mice sustained significantly more weight loss than mice inoculated with 1.6×10^3^ IU of SeV, with a nadir mean weight change from baseline of -28% (p < 0.05). Similarly, by day 5, oxygen saturations were as low as 74% and Penh values were at 7.5-fold over baseline, although these clinical parameters were not statistically different from the other infected cohort (Figure 1).

### Virus replication in the lungs of SeV-infected mice

**Figure 2:**
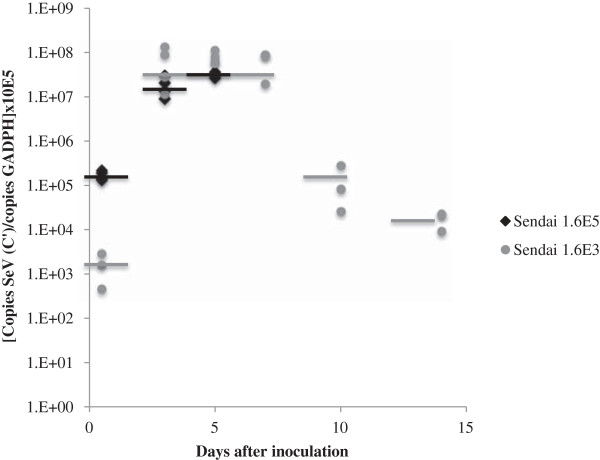
**Virus recovery from mice inoculated with 1.6x10**^**3 **^**or 1.6x10**^**5 **^**IU of Sendai virus.** The day 0 lung virus titers were obtained immediately after inoculation. 1.6E5 and 1.6E3: 1.6x10^5^ 1.6x10^3^ IU of SeV inoculated in 50 μl, respectively.

Viral kinetics from the lungs of DBA/2 mice inoculated with 1.6×10^3^ and 1.6×10^5^ IU of SeV are shown in Figure 2. Despite greater weight loss and higher mortality rate in the mice inoculated with 1.6x10^5^ IU of SeV, there was no difference in virus recovery between the two groups.

### Cellularity of bronchoalveolar lavage fluid from SeV-infected mice

**Figure 3:**
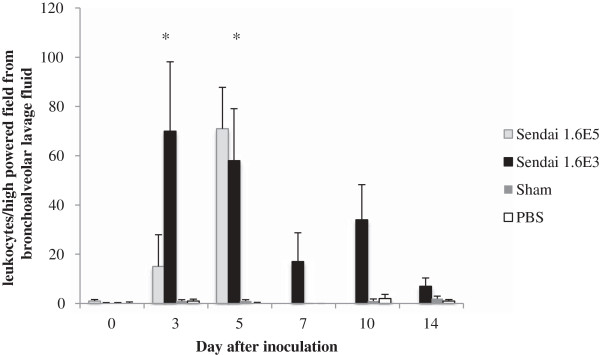
**Cellularity of bronchoalveolar lavage fluid from mice inoculated with PBS, sham stock, 1.6×10**^**3 **^**or 1.6×10**^**5 **^**IU of SeV.** There is statistical difference between the cellularity of bronchoalveolar lavage fluid in SeV-infected mice at either inoculum and the control mice inoculated with either PBS or sham stock at all time points day 3 and later (p < 0.05). Data represent the mean leukocyte number counted from 40 high power fields of cytospin prepared bronchoalveolar fluids from 4 mice per group. * represents statistical difference between cellularity of bronchoalveolar lavage fluid in SeV-infected mice inoculated with 1.6x10^3^ and those inoculated with 1.6x10^5^ IU of SeV.

Pulmonary cellularity of bronchoalveolar lavage fluid from SeV-infected mice is shown in Figure 3. Increased pulmonary cellularity in Sendai infected mice, at both inoculations, is observed on days 3 and 5 followed by a reduction in cellular infiltrate at later time points. This increase in cellularity correlates with both clinical symptoms, such as weight loss and lung function, and production of pro-inflammatory mediators.

### Detection of pro-inflammatory mediators from lungs of SeV-infected mice

**Figure 4:**
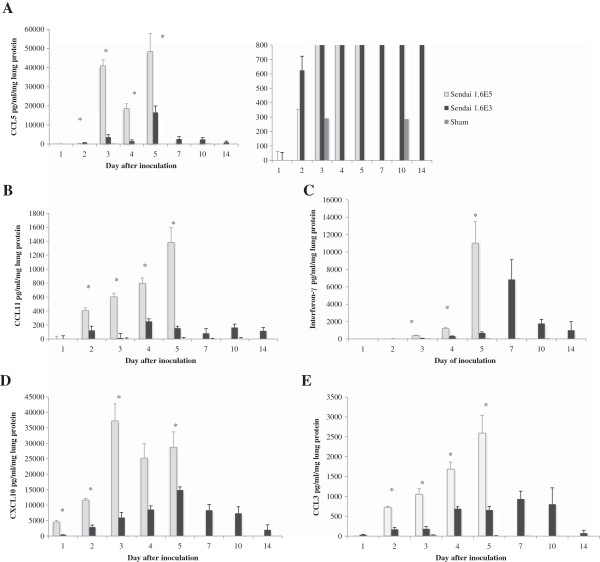
**Detection of pro-inflammatory mediators in lungs of mice inoculated with 1.6×103 or 1.6×105 IU of SeV or sham stock.** There is no data for the mice inoculated with 1.6×105 IU of SeV after day 5 due to death of the mice. **(A)** CCL5. The graph on the left represents the complete data set for CCL5 production in all three groups. The graph on the right is an expanded view of the same data set to easily visualize the difference in CCL5 production between the mice inoculated with 1.6×10^3 and 1.6×10^5 IU of SeV at day 2. **(B)** CCL11 **(C)** Interferon-γ **(D)** CXCL10, **(E)** CCL3 *p < 0.05 when comparing mice inoculated with 1.6x103 and 1.6x105 IU of SeV. At all time points with each inflammatory mediator measured, there is statistical difference between the SeV infected mice and the mice inoculated with sham stock (p<0.05). 1.6E5 and 1.6E3: 1.6x105 1.6x103 IU of SeV inoculated in 50 μl, respectively.

Levels of CCL5, CCL11 (eotaxin), interferon-γ, CXCL10 (interferon gamma-induced protein 10 (IP-10)), and CCL3 (MIP-1α) detected in lung homogenates in response to SeV-infection were significantly higher in the mice inoculated with 1.6×10^5^ IU when compared to those inoculated with 1.6×10^3^ IU (Figure 4; p < 0.05).

**Figure 5:**
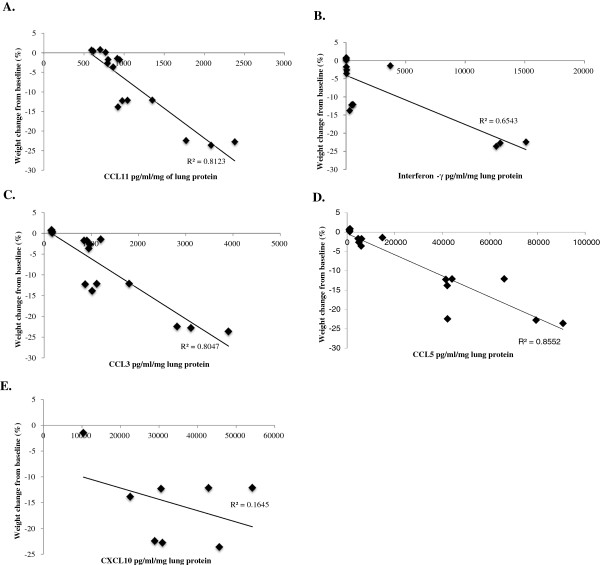
**Scatter plots of concentrations of (A) CCL11, (B) interferon-Υ, (C) CCL3, (D) CCL5, and (E) CXCL10 against weight loss in mice inoculated with 1.6x105 IU of Sendai virus.** Data was collected over 14 days. R2 is the correlation coefficient.

**Figure 6:**
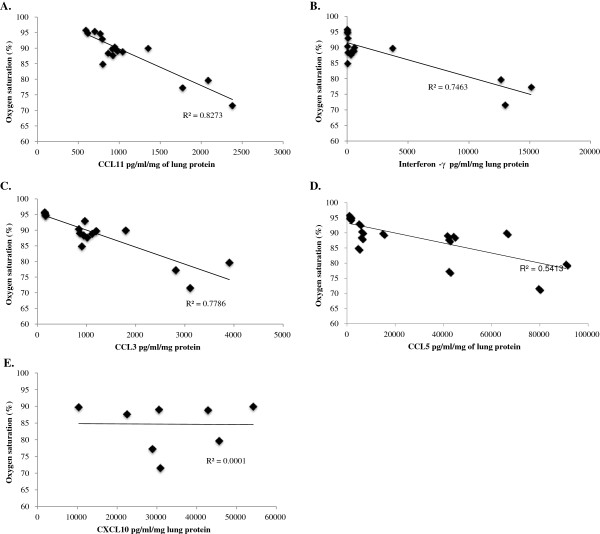
**Scatter plots of concentrations of (A) CCL11, (B) interferon-Υ, (C) CCL3, (D) CCL5, and (E) CXCL10 against percent oxygen saturation in mice inoculated with 1.6x105 IU of Sendai virus.** Data was collected over 14 days. R2 is the correlation coefficient.

**Figure 7:**
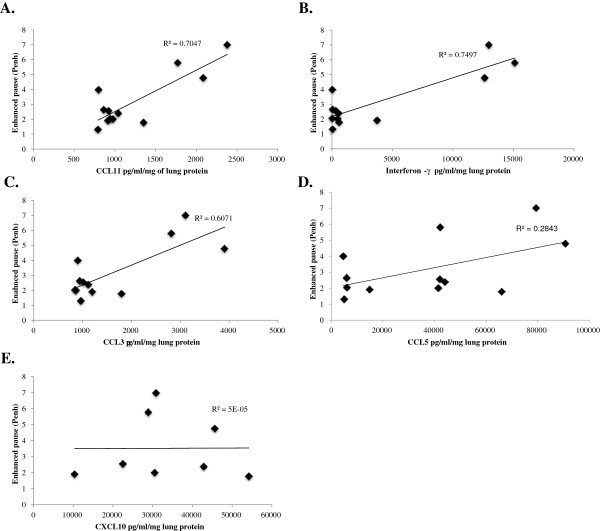
**Scatter plots of concentrations of (A) CCL11, (B) interferon-Υ, (C) CCL3, (D) CCL5, and (E) CXCL10 against Penh values in mice inoculated with 1.6x105 IU of Sendai virus.** Data was collected over 14 days. R2 is the correlation coefficient.

In lethal SeV infection, CCL3, CCL11, and interferon-Υ each strongly correlated with severity of clinical symptoms, including weight loss, diminished oxygen saturation, and Penh (Figures 5, 6 and 7). Interestingly, CCL5 correlated with weight loss (R^2^ = 0.86) and less so with diminished oxygen saturation (R^2^ = 0.54).

On the other hand, in mice inoculated with 1.6×10^3^ IU of SeV, there was no correlation between proinflammatory mediators and clinical parameters. Correlation coefficient (R^2^) for CCL11 and Penh, oxygen saturations, and weight loss were 0.02, 0.006, and 0.09, respectively. R^2^ for IFN-Υ and Penh, oxygen saturations, and weight loss were 0.19, 0.26, and 0.30, respectively. R^2^ for CXCL10 and Penh, oxygen saturations, and weight loss were 0.27, 0.41, and 0.21, respectively. R^2^ for CCL3 and Penh, oxygen saturations, and weight loss were 0.33, 0.44, and 0.59, respectively. R^2^ for CCL5 and Penh, oxygen saturations, and weight loss were 0.13, 0.24, and 0.10, respectively.

Similarly, virus titers did not correlate with clinical parameters at either dose inoculum, with R^2^ of 0.57, 0.58, and 0.25 for Penh, oxygen saturation, and weight loss and inoculum of 1.6×10^5^ IU of SeV, respectively, and R^2^ of 0.12, 0.19, and 0.44 for Penh, oxygen saturation, and weight loss and inoculum of 1.6×10^3^ IU of SeV, respectively (data not in figure).

## Discussion

In this study, we evaluated the clinical impact, virus recovery, and inflammatory responses to both lethal and sublethal SeV infections in inbred DBA/2 mice. As has been previously described, DBA/2 mice are highly susceptible to lethal infection with SeV [11,13-15]. Specifically, we found that DBA/2 mice which received a higher titer virus inoculum (1.6x10^5^ IU of SeV) sustained greater weight loss and mortality than the mice that received the lower inoculum (1.6x10^3^ IU of SeV). Despite the increased severity of disease, virus recovery from mice receiving a lethal dose was not substantially different from that determined for mice receiving the lower virus inoculum. Interestingly, on day 3 post-inoculation, the mice receiving the lower inoculum had statistically higher BAL fluid cellularity when compared to the mice receiving the higher inoculum. The combination of these findings support the observation that severity of illness is not determined by virus recovery alone, and likely results from the combination of direct virus cytotoxicity and the subsequent, host inflammatory response [11,13-15].

The inflammatory response to SeV in the mice differed based on the inoculum. The DBA/2 mice inoculated with 1.6×10^5^ IU of SeV developed a more robust inflammatory response, eliciting higher local levels of CCL11, interferon-γ, CCL3, CCL5, and CXCL10 when compared to mice inoculated with 1.6×10^3^ IU of SeV, despite similar peak virus titers. It is possible that significant differences in early virus kinetics led to the differences in the chemokine response. While this observation is unusual, it is not unique to this study. There are several well-documented parallels demonstrating that there are host- and virus-specific contributions to virus kinetics and resulting chemokine response. Neonatal and adult mice inoculated with the same dose of pneumovirus of mice (PVM), similar to mice of the same strain and age inoculated with either PVM strain 15 or PVM strain J3666, develop differing chemokine responses despite similar virus replication [16,17].

In the lethal infection, CCL11, interferon-γ, and CCL3 each strongly correlated with worsening clinical symptoms, including weight loss, diminished oxygen saturation, and increasing Penh. While interferon- γ and CCL3 are among the mediators identified previously in response to SeV infection [11,12,18], here we correlate production with specific clinical parameters.

CCL11, CCL3, and interferon- γ, are also produced in response to other paramyxovirus infections, including PVM and human RSV infections [19-26]. Furthermore, Jaffri showed that these three pro-inflammatory mediators correlated with worsening histopathologic score and higher Penh values in RSV-challenged mice [25].

Identifying mediators which correlate with disease severity provide targets for therapeutic interventions. In RSV-challenged mice, for example, the use of blocking antibody to CCL11, an eosinophil chemoattractant, resulted in reduced pulmonary eosinophilia and disease severity [23]. Similarly, PVM-infected mice genetically deficient in CCL3 have reduced lung inflammation, although higher lung virus titers, when compared to wild-type mice, suggesting that the inflammatory response induced by CCL3, while potentially harmful to the host, is needed to attenuate virus replication [26]. Treatment of PVM-infected mice with a chemokine receptor antagonist that blocks interaction between CCL3/MIP-1α and its receptor CCR1, in combination with antiviral therapy, results in significant reductions in morbidity and mortality when compared to untreated PVM-infected mice [27].

## Conclusions

We have described here the inflammatory responses to lethal and sublethal inocula in DBA/2 mice and have shown that CCL11, CCL3, and interferon- γ each correlate with disease severity. Further understanding of these responses in the murine model of severe PIV lower respiratory tract infection will aid in the development of therapeutic agents for human disease.

## Methods

### Viral stocks

Six to eight week old DBA/2 mice (Charles River, Wilmington, MA) were intra-nasally inoculated with SeV strain 52, obtained from American Type Culture Collection (ATCC, Manassas, VA). Infected mouse lungs were harvested 5 days post-inoculation, homogenized, pooled, clarified via centrifugation, aliquoted, and stored in liquid nitrogen. All procedures were reviewed an approved by the institution’s Committee for the Humane Use of Animals (CHUA 191). In total, 128 DBA/2 mice were inoculated with 1.6x10^3^ IU of SeV over 7 experiments and 52 DBA/2 mice were inoculated with 1.6×10^5^ IU of SeV over 3 experiments to collect the following data.

### Sham stocks

Lungs from uninfected 6–8 week old DBA/2 mice purchased from Charles River (Wilmington, MA) were harvested 5 days post-inoculation, homogenized, pooled, clarified via centrifugation, aliquoted, and stored in liquid nitrogen and used as sham stock control.

### Establishing infection in mice

Specific pathogen-free, 5–8 week old DBA/2 mice were purchased from Charles River (Wilmington, MA). Virus stocks of mouse passaged SeV stored in liquid nitrogen were diluted in phosphate buffered saline (PBS) to final concentrations of 1.6×10^3^ and 1.6×10^5^ infectious units (IU)/50 μl. Under light isofluorane anesthesia, the DBA/2 mice were intranasally inoculated with either 1.6×10^3^ or 1.6×10^5^ IU in 50 μl. Control mice received either an intra-nasal dose of PBS diluent or sham stock (50 μl).

Clinical symptoms, as per scoring system previously described, together with body weights, and pulse oximetry were recorded daily [28]. Whole body plethysmography (WBP) was performed daily for a 5-minute period, as previously described, and measured enhanced pause (Penh) values were recorded [28,29]. Oxygen saturations were measured daily using a throat clip pulse oximeter (Starr Life Sciences Corp, Oakmont, PA). One day prior to inoculation, hair at the site of the oximeter sensor was removed using a depilatory cream. At the time of daily oximetry measurements, the appropriate-sized throat clip was applied to the unanesthetized mouse. Pulse oximetry, heart rates, and respiratory rates were recorded.

### Determination of virus titers in SeV-infected mouse lung tissue

Virus titers were determined on days 0, 3, 5, 7, 10, and 14 after intranasal inoculation with SeV by a quantitative reverse transcriptase PCR assay that targets the SeV C’ gene. Lung tissue was excised from SeV-infected mice, homogenized in RNazol B and RNA prepared per manufacturer’s instructions. Integrity of the RNA was evaluated on formaldehyde agarose gels and quantified spectrophotometrically (A_260_/A_280_). cDNA was prepared from 2 μg DNase I-treated RNA from each lung sample and subjected to quantitative RT-PCR using primers and probe targeting the virus C’ gene: probe: 5′-56FAM-ACTTCTCCTTCGCCCTCACTT 36TAMSp-3′, primer 1: 5′-AACACCAATCAACACTCCCC-3′, primer 2: 5′-CCTGATCGATTATCTTGGGTC-3′, with reference to a standard curve generated using serial ten-fold dilutions of the C’ gene as target. The cDNAs were then subjected to a second set of quantitative PCR assays, with the commercial rodent GAPDH primers and probe (Applied Biosciences, Foster City, CA) and serial ten-fold dilutions of the 905 bp rodent GAPDH fragment (Applied Biosciences, Foster City, CA) to generate a standard curve. Controls include reactions with no reverse transcriptase and no template. Reactions were performed in an ABI 7500 Sequence detector, with cycling parameters including: 50°C for 2 min, 95°C for 10 min, followed by 40 cycles of 95°C for 15 s and 65°C for 1 min. Data are expressed as copies of C’ per 10^5^ copies GAPDH.

### Bronchoalveolar lavage fluid and cell counts

At given time points, mice were euthanized (four mice per condition per time point), and bronchoalveolar lavage (BAL) fluid was harvested by transtracheal instillation of pre-chilled PBS with 2% EDTA, with recovery of approximately 700 μl of BAL fluid. Leukocyte counts were obtained by visual inspection and quantitative analysis cytospin preparations that were stained following Fisher Scientific Hema 3 protocol.

### Determination of concentrations of proinflammatory mediators and growth factors from SeV-infected mouse lungs

Concentrations of proinflammatory mediators, including CCL5, CCL11, interferon-γ, CXCL10, and CCL3 were determined on days 0, 1, 2, 3, 4, 5, 7, 10, and 14 on total lung homogenates from SeV- , sham stock-, and diluent control-inoculated mice (four mice each per time point) using commercially-available ELISA kits (R&D Systems, Minneapolis, MN).

### Statistical analysis

Data were analyzed using ANOVA or Student t-test’s as appropriate.

## Competing interests

The authors have no competing interests to declare.

## Authors’ contributions

MS participated in the design of the study, performing experiments, data analysis and interpretation, and drafted the manuscript. CB participated in the design of the study and performing experiments. HR participated in the design of the study, interpretation of the data, and critical review of the manuscript. JD participated in the design of the study, data analysis and interpretation, and critical review of the manuscript. All authors have read and approved the final manuscript.
